# Association between Live Dietary Microbes and Root Caries: A Cross-Sectional Study with a Predictive Risk Model

**DOI:** 10.3290/j.ohpd.c_2532

**Published:** 2026-02-24

**Authors:** Donglei Wu, Zhengshen Lin, Peng Zhou, Hongxia You, Weixuan Chen, Yuyan Zheng

**Affiliations:** a Donglei Wu# Dentist, Department of Stomatology, Shenzhen People’s Hospital, Shenzhen, China. Study design, conducting the study, analysing the data, writing and reviewing the manuscript.; b Zhengshen Lin# Dentist, Department of Stomatology, The People’s Hospital of Baoan Shenzhen, The Second Affiliated Hospital of Shenzhen University, Shenzhen, China. Study design, conducting the study, analysing the data, writing and reviewing the manuscript.; c Peng Zhou Dentist, Department of Stomatology, Guangdong Women’s and Children’s Hospital, Guangzhou, Guangdong, China. Study design, conducting the study, analysing the data, writing and reviewing the manuscript.; d Hongxia You Dentist, Department of Stomatology, Shenzhen People’s Hospital, Shenzhen, China. Study design, conducting the study, analysing the data, writing and reviewing the manuscript.; e Weixuan Chen Nurse, Department of Stomatology, Shenzhen People’s Hospital, Shenzhen, China. Conducting the study, analysing the data.; f Yuyan Zheng Dentist, Department of Stomatology, Shenzhen People’s Hospital, Shenzhen, China. Study design, conducting the study, analysing the data, writing and reviewing the manuscript. # These authors contributed equally to this work.

**Keywords:** cross-sectional study, dietary habits, epidemiology, live dietary microbes, oral biofilms, root caries

## Abstract

**Purpose:**

To investigate the association between medium‑to‑high (MedHi) levels of live dietary microbe intake and the prevalence of root caries, and to develop a predictive model for estimating root caries risk.

**Methods and Materials:**

This cross‑sectional study analysed data from participants in the National Health and Nutrition Examination Survey (NHANES) 2015–2020. Dietary intake was assessed using 24‑h dietary recalls, and root caries status was determined via standardised oral examinations. Univariate and multivariable logistic regression analyses were conducted to identify factors associated with root caries, including live dietary microbe intake. Participants were randomly divided into training and testing data sets. A least absolute shrinkage and selection operator (LASSO) regression was used to construct a predictive model, which was visualised using a nomogram and evaluated by receiver operating characteristic (ROC) curves and calibration plots.

**Results:**

Among 7,839 participants, MedHi live dietary microbe intake, age, education level, smoking status, dental floss frequency, and systemic conditions were significantly associated with root caries (P < 0.05). The predictive model incorporating these variables demonstrated good discrimination and calibration in both the training and testing data sets.

**Conclusion:**

Higher intake of live dietary microbes was associated with a lower prevalence of root caries. Although the cross‑sectional design precludes causal inference, the findings suggest a potential link between dietary microbes and oral health. The proposed model may aid clinicians in identifying individuals at high risk and in developing targeted preventive strategies.

As life expectancy increases and populations age worldwide, the prevalence of dental root caries has become a growing concern in recent decades.^10^ The rising number of new root caries cases among older adults is anticipated to present one of the major challenges in contemporary dentistry. Recent US estimates (2017–2020) report an untreated root caries prevalence above 10% among adults 40 years and older.^2,13^ Dental root caries refers to the demineralisation and destruction of the tooth root surface that occurs below the cementoenamel junction (CEJ), excluding the adjacent enamel. It is a multifactorial infectious disease initiated primarily by cariogenic bacteria and influenced by local and systemic factors. Root caries can cause pain, tooth loss, and functional impairment, thereby compromising general health and diminishing the quality of life in older adults. Maintaining microbial balance in the oral cavity plays an important role in preventing dental diseases. Live dietary microbes, such as those present in fermented foods and probiotic products, may contribute to oral microbial homeostasis by modulating the growth of pathogenic bacteria. Recent research has renewed interest in the relationship between live dietary microbes and the prevention of root caries. Therefore, the present study aimed to investigate the correlation between the intake of live dietary microbes and the prevalence of dental root caries, and to develop a practical, machinelearning-based predictive model for assessing root caries risk among adults.

## METHODS AND MATERIALS

### Data Resources

This cross‑sectional study utilised data from the 2015–2020 National Health and Nutrition Examination Survey (NHANES), a programme conducted by the National Centre for Health Statistics (NCHS) of the US Centers for Disease Control and Prevention. NHANES is designed to assess the health and nutritional status of non-institutionalised, community‑dwelling adults and children in the United States, providing a nationally representative sample of the US population. The NHANES protocol was approved by the NCHS Research Ethics Review Board (available at https://wwwn.cdc.gov/Nchs/Nhanes/). Written informed consent was obtained from all participants prior to data collection. All methods were performed in accordance with relevant guidelines and regulations.

### Study Population

**Fig 1:**
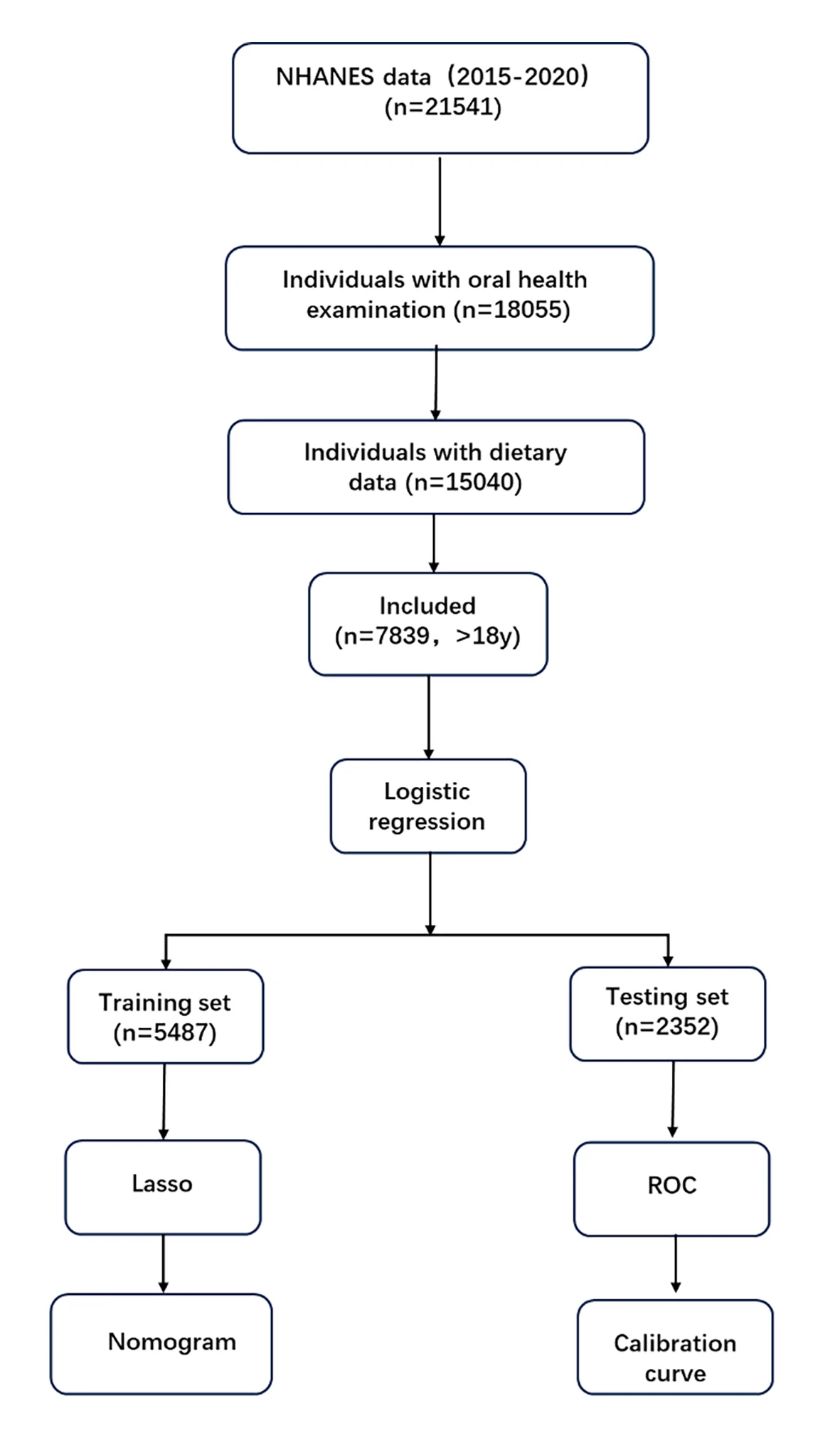
Flowchart of the study population

During the 2015–2020 NHANES cycle, data from 21,541 participants were available. Participants were included if they had complete oral health examination data and dietary intake data, including information on live dietary microbes, dairy products, sugar‑sweetened beverages (SSBs), fruits, vegetables, protein foods, and grains. Individuals younger than 19 years or with missing values were excluded (n = 7201). After applying these criteria, 7839 participants remained for analysis (Fig 1). Among them, 6419 participants were classified into the non-root-caries-experience group and 1420 into the root-caries-experience group.

### Dietary Live Microbes

Foods containing live microorganisms were categorised into low, medium (Med), and high (Hi) groups based on a recent classification system derived from 24‑h dietary recall data.^15^ Foods with fewer than 10^4^ CFU/g of living microbes, primarily heat‑treated or processed products, were classified as having a low level of live microbes. Those with microbial counts ranging between 10^4^ and 10^7^ CFU/g were classified as medium‑level foods, mainly consisting of raw fruits and vegetables. Foods with microbial counts exceeding 10^7^ CFU/g, such as unpasteurised fermented foods and probiotic supplements, were classified as high‑level foods.^15^ Each dietary live microbe variable was recorded as a continuous variable, and a combined ‘medium‑to‑high’ (MedHi) category was created by aggregating participants who consumed foods from both the Med and Hi categories. For analytical purposes, the intake of low‑level live microbes was divided into four quartiles (Q1–Q4): Q1 (median = 1675.07 g/d, range = 230.79–2134.84 g/d); Q2 (median = 2513.43 g/d, range = 2134.84–2913.25 g/d); Q3 (median = 3322.71 g/d, range = 2913.25–3895.57 g/d); and Q4 (median = 4815.33 g/d, range = 3895.57–18 281.03 g/d). Similarly, MedHi dietary live microbe intake was categorised into four quartiles: Q1 (median = 0 g/d; no intake of MedHi dietary live microbes); Q2 (median = 20 g/d, range = 0–35.5 g/d); Q3 (median = 90 g/d, range = 35.6-170 g/d); and Q4 (median = 286.88 g/d, range = 170–1824.32 g/d).

### Root Caries

Evaluation for the presence of root caries and root restorations was limited to teeth exhibiting gingival recession. In each quadrant showing root exposure, the tooth surfaces were air‑dried and examined using a surface‑reflecting mirror and a No. 23 explorer. A lesion was diagnosed as root caries if demineralisation on the root surface extended at least 1 mm apically beyond the cementoenamel junction (CEJ) or if more than 50% of the lesion area was on the root surface adjacent to the CEJ. Data were recorded as the overall presence or absence of root caries and root restorations. Detailed information on the NHANES dental examination and root caries detection procedures is available at https://www.cdc.gov/nchs/nhanes.

### Other Covariables

Demographic characteristics of participants, including age, gender, ethnicity, and education level, were obtained from the NHANES questionnaire. Hypertension was defined as blood pressure greater than 140/90 mmHg. Diabetes mellitus (DM) was defined as glycohemoglobin (HbA1c) > 6.5%, fasting plasma glucose ≥ 7.0 mmol/L, random or 2-h oral glucose tolerance test (OGTT) plasma glucose ≥ 11.1 mmol/L, or current use of antidiabetic medication or insulin therapy. Parkinson’s disease was identified based on the reported use of antiparkinsonian agents. Smoking status was classified as a current smoker or a non‑smoker. Oral hygiene habits were assessed based on the number of days per week participants reported using dental floss. Dietary intake data were derived from the first‑day 24‑h dietary recall interview and treated as continuous variables. The dietary components analysed included live dietary microbes, sugar‑sweetened beverages (SSBs), dairy products, protein foods, vegetables, fruits, and grains. SSBs included fruit drinks with less than 100% juice, sports and energy drinks, nutritional beverages, smoothies, grain‑based drinks, bottled or carbonated water, and sweetened coffee or tea. Dairy products encompass milk, yoghurt, and cheese. Protein sources included meats (beef, pork, veal, fish, lamb, and game), eggs, soy‑based products, nuts, seeds, and legumes.

### Statistical Analysis

Continuous variables, including age and dietary intake, were summarised as medians with interquartile ranges (IQRs), while categorical variables, such as gender, ethnicity, education level, smoking status, and history of diabetes, hypertension, and Parkinson’s disease, were presented as numbers (percentages). Missing data were handled using multiple imputation by chained equations (MICE); ten imputed data sets were generated, and the pooled estimates were derived according to Rubin’s rules. Comparisons of non‑normally distributed continuous variables between participants with and without root caries were performed using the Kruskal–Wallis test, whereas categorical variables were compared using the chi‑square test. Univariate and multivariate logistic regression analyses were conducted to assess the independent association between root caries experience and dietary live microbe intake, and results were expressed as odds ratios (ORs) with corresponding 95% confidence intervals (CIs). Three multivariate logistic regression models were constructed. Model 1 was adjusted for age, gender, ethnicity, education level, and intake of low‑level live microbes. Model 2 was further adjusted for intake of fruits, vegetables, dairy products, grains, protein foods, SSBs, days of using dental floss per week and smoking status. Model 3 was additionally adjusted for hypertension, diabetes mellitus (DM), and Parkinson’s disease. A two‑tailed P value < 0.05 was considered statistically significant for all analyses.

### Model Construction, Evaluation, and Validation for Root Caries Diagnosis by Machine Learning

To develop a predictive model for root caries, all participants were randomly divided into two subsets – the training and testing data sets – at a ratio of 7:3 using the ‘caret’ package in R. The training set was used for model development, whereas internal validation was conducted on the testing set. Least absolute shrinkage and selection operator (LASSO) regression analysis was performed using the ‘glmnet’ package in R to identify the most informative predictors, and a corresponding nomogram model was subsequently constructed with the ‘rms’ package. The penalty parameter (λ) was tuned via 10‑fold cross‑validation and selected according to the one‑standard‑error rule (λ₁SE), ensuring a parsimonious and stable model; as a sensitivity analysis, the solution at λmin, which retains more predictors, was also examined. Model performance was evaluated using receiver operating characteristic (ROC) curve analysis, and the area under the curve (AUC) was calculated to quantify discriminative ability. Calibration curves were generated to assess the agreement between the model‑predicted probabilities and the actual observed outcomes.

## RESULTS

### Basic Characteristics of Participants

A total of 7839 participants met the inclusion criteria. Significant differences were observed between individuals with and without root caries experience in the distribution of demographic factors – including age, gender, ethnicity, and education level – as well as in dietary intake variables (vegetables, grains, protein foods, and MedHi live dietary microbes), smoking status, days of flossing per week, and systemic conditions such as diabetes mellitus, hypertension, and Parkinson’s disease (P < 0.05) (Table 1).

**Table 1:** Demographic characteristics of US by the National Health and Nutrition Examination Survey 2015–2020

Variable	Total	Non-root caries experience (n = 6419)	Root caries experience (n = 1420)	P value
**Age**				**< 0.0001**
Median (IQR)	46.00 (31.00, 60.00)	44.00 (30.00, 58.00)	56.00 (43.00, 66.00)	
**Gender, n (%)**				**0.03**
Female	4055 (51.11)	3427 (51.87)	628 (47.12)	
Male	3784 (48.89)	2992 (48.13)	792 (52.88)	
**Ethnicity, n (%)**				**< 0.001**
Black	1785 (11.19)	1409 (10.89)	376 (12.80)	
Mexican	1210 (9.33)	1022 (9.68)	188 (7.52)	
White	2753 (63.26)	2184 (62.32)	569 (68.18)	
Other	2091 (16.21)	1804 (17.11)	287 (11.50)	
**Education, n (%)**				**< 0.0001**
>12th grade	4542 (64.72)	3891 (67.27)	651 (51.37)	
9th–12th grade	2731 (31.91)	2095 (29.55)	636 (44.29)	
< 9th grade	566 (3.37)	433 (3.18)	133 (4.34)	
**Low live microbes (g)**				**0.42**
Q1	1960 (20.05)	1571 (19.65)	389 (22.14)	
Q2	1960 (23.69)	1617 (23.88)	343 (22.67)	
Q3	1959 (27.12)	1621 (26.98)	338 (27.82)	
Q4	1960 (29.14)	1610 (29.48)	350 (27.37)	
**MedHi live microbes (g)**				**< 0.001**
Q1	2910 (32.39)	2243 (30.34)	667 (43.14)	
Q2	1010 (13.14)	848 (13.34)	162 (12.12)	
Q3	1973 (26.90)	1643 (27.44)	330 (24.05)	
Q4	1946 (27.57)	1685 (28.88)	261 (20.69)	
**Fruit (cup)**				**< 0.0001**
Median (IQR)	0.35 (0.00, 1.36)	0.42 (0.00, 1.39)	0.09 (0.00, 1.19)	
**Vegetable (cup)**				**< 0.001**
Median (IQR)	1.26 (0.59, 2.18)	1.29 (0.63, 2.24)	1.09 (0.48, 1.83)	
**Grain (oz)**				**0.03**
Median (IQR)	5.81 (3.61, 8.53)	5.89 (3.69, 8.64)	5.48 (3.39, 8.20)	
**Protein foods (oz)**				**0.02**
Median (IQR)	5.46 (3.19, 8.53)	5.52 (3.29, 8.63)	4.96 (2.83, 8.00)	
**Dairy (cup)**				**0.07**
Median (IQR)	1.14 (0.49, 2.11)	1.15 (0.50, 2.15)	1.09 (0.44, 1.92)	
**SSB (g)**				**0.01**
Median (IQR)	184.00 (0.00, 558.00)	161.00 (0.00, 527.00)	264.50 (0.00, 667.00)	
**Smoking status, n (%)**				**< 0.0001**
No	4763 (60.11)	4164 (64.09)	599 (39.23)	
Yes	3076 (39.89)	2255 (35.91)	821 (60.77)	
**Diabetes, n (%)**				**< 0.0001**
No	5723 (77.88)	4821 (79.50)	902 (69.41)	
PreDM	632 (7.72)	512 (7.69)	120 (7.89)	
DM	1484 (14.40)	1086 (12.81)	398 (22.70)	
**Hypertension, n (%)**				**< 0.001**
No	4523 (63.53)	3916 (66.45)	607 (48.25)	
Yes	3316 (36.47)	2503 (33.55)	813 (51.75)	
**Parkinson, n (%)**				**0.04**
No	7768 (99.15)	6369 (99.28)	1399 (98.49)	
Yes	71 (0.85)	50 (0.72)	21 (1.51)	
**Days of flossing per week**				
	3.00 (0.00, 7.00)	3.00 (0.00, 7.00)	2.00 (0.00, 7.00)	**0.002**
SSB, sugar-sweetened beverages; IQR, interquartile range; PreDM, prediabetes, which includes impaired fasting glucose (IFG) or impaired glucose tolerance (IGT).				

### Univariate Regression Analysis for the Association Between Root Caries and Live Microbes

Univariate logistic regression analysis showed that Mexican ethnicity, as well as higher intakes of fruits, vegetables, grains, days of flossing per week, and MedHi live dietary microbes, were negatively associated with the presence of root caries (P < = 0.05). In contrast, older age, male, lower education level, smoking, and the presence of systemic diseases – including hypertension, diabetes mellitus (DM), and Parkinson’s disease – were positively associated with root caries (P < 0.05) (Table 2).

**Table 2:** Univariate regression analysis for the association between root caries and live microbes

Variable	Estimate	Std. Error	t value	Pr(>|t|)	OR	95% CI
**Age**						
< =30	ref	ref	ref	ref	ref	ref
30–60	1.02	0.16	6.49	< 0.0001	2.78	(2.01, 3.83)
>60	1.53	0.15	10.03	< 0.0001	4.6	(3.37, 6.29)
**Gender**						
Female	ref	ref	ref	ref	ref	ref
Male	0.19	0.08	2.36	0.03	1.21	(1.03, 1.43)
**Ethnicity**						
Black	ref	ref	ref	ref	ref	ref
Mexican	–0.42	0.19	–2.2	0.04	0.66	(0.45, 0.97)
Other	–0.56	0.11	–5.16	< 0.0001	0.57	(0.46, 0.71)
White	–0.07	0.11	–0.66	0.51	0.93	(0.74, 1.16)
**Education**						
>12th grade	ref	ref	ref	ref	ref	ref
9th–12th grade	0.67	0.12	5.64	< 0.0001	1.96	(1.54, 2.51)
< 9th grade	0.58	0.14	4.07	< 0.001	1.79	(1.33, 2.39)
**Low live microbes (g)**						
Q1	ref	ref	ref	ref	ref	ref
Q2	–0.17	0.12	–1.39	0.18	0.84	0.84 (0.65, 1.08)
Q3	–0.09	0.14	–0.65	0.52	0.91	0.91 (0.69, 1.21)
Q4	–0.19	0.1	–1.99	0.06	0.82	0.82 (0.67, 1.01)
**MedHi live microbes (g)**						
Q1	ref	ref	ref	ref	ref	ref
Q2	–0.448	0.165	–2.706	0.012	0.64	(0.46, 0.90)
Q3	–0.484	0.148	–3.258	0.003	0.62	(0.46, 0.84)
Q4	–0.686	0.181	–3.792	< 0.001	0.50	(0.35, 0.73)
**Fruit (cup)**	–0.09	0.04	–2.45	0.02	0.91	(0.85, 0.99)
**Vegetable (cup)**	–0.18	0.05	–3.86	< 0.001	0.83	(0.75, 0.92)
Grain (oz)	–0.02	0.01	–2.16	0.04	0.98	(0.95, 1.00)
Protein foods (oz)	–0.02	0.01	–1.82	0.08	0.98	(0.96, 1.00)
Dairy	–0.05	0.03	–1.59	0.12	0.95	(0.88, 1.02)
**SSB (g)**	0	0	3.6	0.001	1	(1.00, 1.00)
**Smoking status**						
No	ref	ref	ref	ref	ref	ref
Yes	1.02	0.08	13.33	< 0.0001	2.77	(2.37, 3.23)
**Hypertension**						
No	ref	ref	ref	ref	ref	ref
Yes	0.75	0.09	8.62	< 0.0001	2.12	(1.78, 2.54)
**DM**						
No	ref	ref	ref	ref	ref	ref
PreDM	0.16	0.17	0.95	0.35	1.18	(0.83, 1.67)
DM	0.71	0.13	5.52	< 0.0001	2.03	(1.56, 2.64)
**Parkinson**						
No	ref	ref	ref	ref	ref	ref
Yes	0.75	0.35	2.11	0.04	2.11	(1.02, 4.34)
**Days of flossing per week**	–0.06	0.02	–3.07	0.05	0.94	0.94 (0.90, 0.98)


### Multivariable Logistic Regression for the Association Between Root Caries and MedHi Live Microbes

Table 3 summarises the associations between root caries and the intake level of MedHi dietary live microbes in the multivariable logistic regression analysis. In the unadjusted model, a significant inverse relationship was observed between the consumption of MedHi live dietary microbes and the prevalence of root caries (P for trend < 0.001). After adjustment for age, gender, ethnicity, education level, and intake of low live microbes in Model 1, the intake of MedHi live dietary microbes remained negatively associated with root caries compared with non-intake (Q1) (P < 0.05). Increasing consumption of MedHi live dietary microbes was associated with progressively lower odds ratios (ORs) for root caries, as shown in Table 3 (P for trend < 0.05). Similarly, in Model 2 (adjusted for Model 1 plus intake of fruits, vegetables, dairy products, grains, protein foods, SSBs, days of using dental floss per week, and smoking status), the negative association persisted, particularly in the Q3 and Q4 quartiles (P for trend < 0.05). After further adjustment for all potential confounders in Model 3 (including hypertension, DM, and Parkinson’s disease), only the Q3 quartile of MedHi live dietary microbe intake remained significantly inversely associated with root caries (OR = 0.69, 95% CI: 0.49–0.97; P < 0.05; P for trend < 0.05), when compared with the nonintake group (Q1).

**Table 3:** Multiple logistic regression analysis for the association between root caries and intake of MedHi live microbes

MedHi live microbes	Multivariate logistic regression analysis			
	Crude model	Model 1	Model 2	Model 3
Character	OR (95%CI) P value	OR (95%CI) P value	OR (95%CI) P value	OR (95%CI) P value
Q1 [0, 0]	1.00 Ref	1.00 Ref	1.00 Ref	1.00 Ref
Q2 (0, 35.5]	0.64 (0.46, 0.90) 0.01	0.68 (0.48, 0.97) 0.03	0.71 (0.49, 1.02) 0.06	0.71 (0.47, 1.05) 0.08
Q3 (35.5, 170]	0.62 (0.45, 0.84) 0.003	0.61 (0.45, 0.82) 0.003	0.69 (0.50, 0.94) 0.02	0.69 (0.49, 0.97) 0.04
Q4 (170, 1824.32]	0.50 (0.35, 0.73) < 0.001	0.51 (0.35, 0.73) 0.001	0.64 (0.43, 1.01) 0.05	0.66 (0.42, 1.04) 0.07
P for trend*	< 0.001	< 0.001	0.02	0.03
				

Model 1 was adjusted for age, gender, ethnicity, education level, and intake of low live microbes.

Model 2 was adjusted for age, gender, ethnicity, education level, intake of low live microbes, fruits, vegetables, dairy products, grains, protein foods, SSBs, smoking status, and days of dental floss use per week.

Model 3 was adjusted for age, gender, ethnicity, education level, intake of low live microbes, fruits, vegetables, dairy products, grains, protein foods, SSBs, smoking status, hypertension, DM, days of dental floss use per week, and Parkinson’s disease. OR, odds ratio; CI, confidence interval; Q1, quartile 1 (median = 0 g/d); Q2, quartile 2 (median = 20 g/d); Q3, quartile 3 (median = 90 g/d); Q4, quartile 4 (median = 286.88 g/d).

### LASSO Regression Analysis to Identify Key Dietary Factors for Root Caries

**Fig 2a and b:**
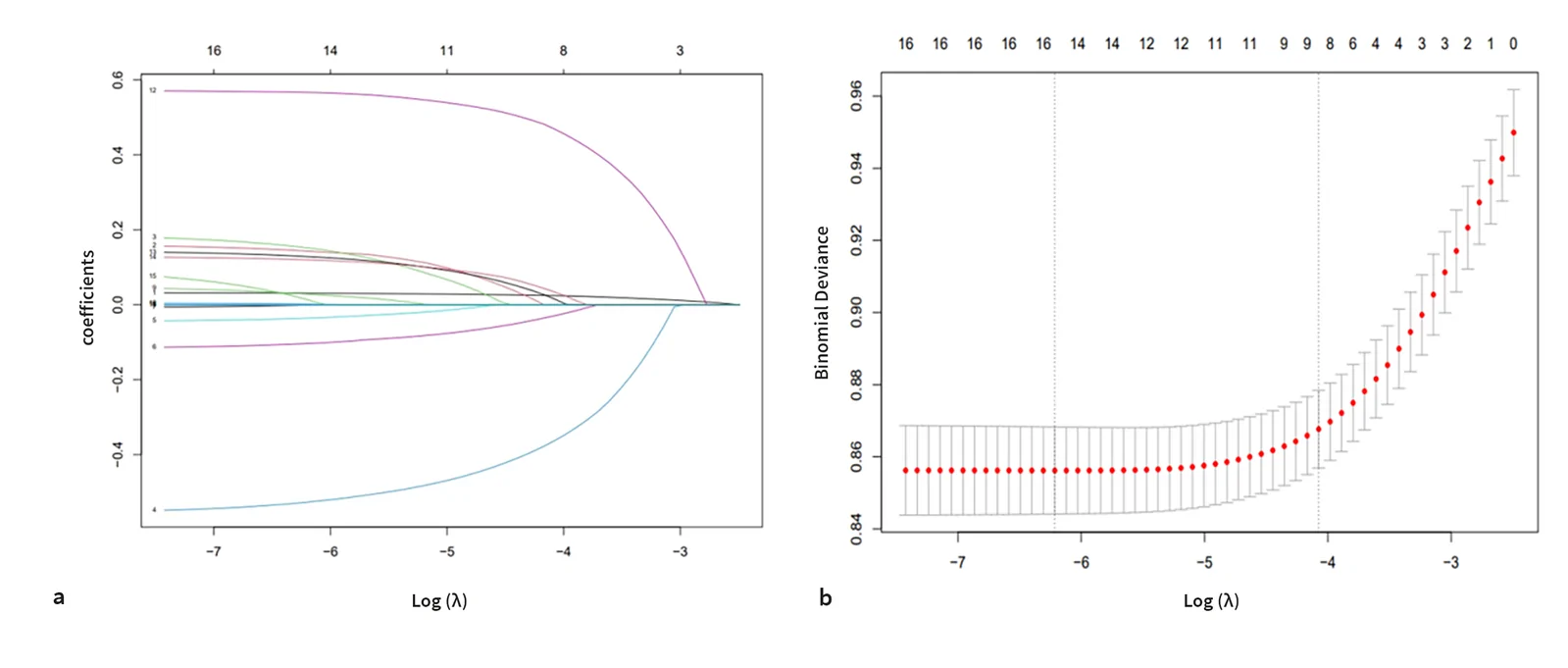
LASSO regression analysis for identifying key dietary factors associated with root caries. *(a) *Coefficient profiles of the variables from the LASSO regression. The dotted vertical line indicates the optimal λ value selected via tenfold cross-validation. Nine variables with non-zero coefficients were retained for model construction. *(b) *Tenfold cross-validation plot of the LASSO model. The mean squared error (MSE) of cross-validation is plotted against the log (λ). The yaxis represents the MSE, and the xaxis indicates the log-transformed λ values.

**Fig 3a to d:**
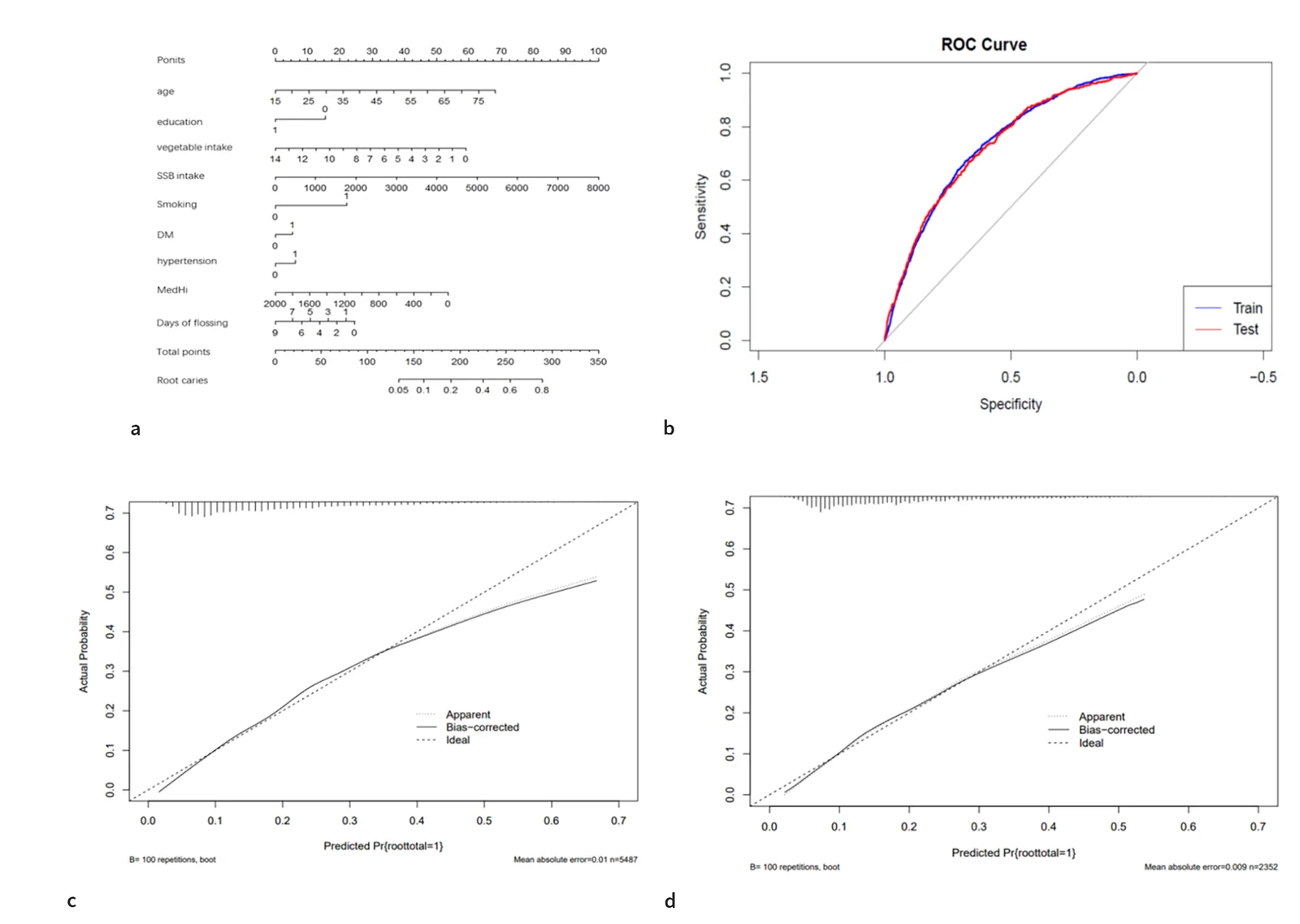
Nomogram model for predicting the risk of root caries and model validation. *(a) *Nomogram constructed using key dietary and clinical factors identified through LASSO regression. For each predictor, draw a vertical line upward to the ‘Points’ axis to determine its individual score. The total points, obtained by summing the scores of all predictors, correspond to the predicted probability of root caries shown at the bottom of the nomogram. Education: 0 = ≤ 12th grade; 1 = > 12th grade. Smoking: 0 = nonsmoker; 1 = smoker. Hypertension: 0 = no; 1 = yes. DM: 0 = no diabetes; 1 = diabetes. *(b) *Receiver operating characteristic (ROC) curves of the nomogram model for the training and testing data sets. The x-axis represents specificity, and the y-axis represents sensitivity. The area under the ROC curve (AUC) was calculated to evaluate the discriminatory performance of the model. *(c) *Calibration plot of the nomogram in the training data set. *(d) *Calibration plot of the nomogram in the testing data set. The x-axis shows the predicted probability of root caries according to the nomogram, while the y-axis represents the observed probability.

A total of 7839 participants were randomly divided into two data sets: 5487 for model training and 2352 for testing. Using the least absolute shrinkage and selection operator (LASSO) regression, nine optimal variables were selected from an initial set of 17 predictors (Fig 2). These selected variables were then incorporated into a multivariable logistic regression model to construct a predictive model for root caries. The final model identified nine statistically significant predictors: intake of MedHi live dietary microbes, age, education level, vegetable intake, SSB intake, smoking status, days of dental floss use per week, DM, and hypertension. These independent predictors were subsequently integrated into a nomogram to quantify the individual risk of developing root caries (Fig 3). As a sensitivity analysis, the solution at λmin, which retained 15 predictors, was also examined; however, its discrimination was very similar to that of the λ₁SE-based model.

### Construction of a New Predictive Model for Root Caries

A nomogram model was constructed to predict the risk of root caries based on the nine selected variables. The ROC curve analysis indicated satisfactory predictive performance, with the area under the curve (AUC) reaching 0.728 in the training data set and 0.726 in the testing data set (Fig 3). The calibration plots demonstrated good agreement between the predicted and observed probabilities in both data sets, showing strong calibration with a mean absolute error of < 0.05, mean squared error of < 0.05, and the 0.9 quantile of absolute error of < 0.05 (Fig 3). As a sensitivity analysis, a more complex model derived from the λmin solution and including 15 predictors yielded very similar discrimination (training AUC 0.729 and testing AUC 0.724), indicating that the predictive performance was robust to the choice of λ (Supplementary Fig 1).

## DISCUSSION

Although various non‑invasive and invasive approaches have been developed to arrest the progression of root caries, clinical studies have consistently reported a high failure rate of root-surface restorations. Loss of retention remains the most common cause of restoration failure, followed by secondary caries.^1^ Furthermore, root caries exerts a bidirectional effect, compromising the tooth surface while simultaneously impairing the surrounding periodontal tissues-thereby amplifying the overall impact on an individual’s oral health.^1^ Consequently, strategies emphasising prevention and early detection of root caries are more practical and essential than those focusing solely on restorative interventions.

This cross-sectional study identified an inverse association between the intake of MedHi live dietary microbes and the prevalence of root caries among adults. Individuals who consumed more foods rich in live microorganisms showed a lower probability of developing root caries. In addition, we developed a multivariable risk prediction model incorporating demographic, dietary, and systemic disease-related variables. To our knowledge, this is the first study to include live dietary microbes as a component in a machine‑learning-based prediction model for root caries risk.

Our findings align with previous evidence suggesting that probiotics and fermented foods contribute to oral health maintenance and caries prevention. Several studies have reported that probiotics such as *Lactobacillus*, *Bifidobacterium*, and *Streptococcus* species can reduce the salivary count of *S. mutans*, the primary cariogenic microorganism. Likewise, randomised trials have demonstrated that probiotic supplementation or fermented dairy consumption reduces bacterial colonisation and improves salivary protective factors in elderly populations. On the other hand, some systematic reviews found no significant effect of probiotic intake on salivary microbiota composition, highlighting variability in strain specificity, dosage, and intervention duration. These mixed results indicate the need for more standardised, mechanistic research.

### Biological Plausibility and Mechanistic Explanations

The protective effect of live dietary microbes against root caries can be biologically explained through several interrelated mechanisms supported by previous evidence. First, microbial competition and biofilm modulation play central roles. Probiotics compete with cariogenic bacteria such as *S. mutans* for adhesion sites and nutrients, thus interfering with biofilm formation.^21^ Enzymes and antimicrobial metabolites produced by probiotics, including mutanase and dextranase, can degrade extracellular polysaccharides within dental plaque, further hindering bacterial aggregation.^21^ Second, acid production and pH regulation are critical determinants of mineral dissolution. While pathogenic biofilms lower local pH and promote irreversible demineralisation,^5^ probiotic bacteria can help stabilise oral pH by producing mild organic acids and enhancing salivary buffering capacity.^9,14^ Fermented milk products, for example, have been reported to reduce enamel mineral loss in vitro and decrease cariogenic microorganisms within the oral cavity.^14^ Third, host–microbe interactions and immune modulation offer another explanation. Fermented foods rich in live microbes can strengthen mucosal immunity and improve salivary defence factors, thereby reducing susceptibility to microbial dysbiosis.^6,16^ A recent animal study also found that fermented plant foods, such as Japanese mugwort, altered the oral microbiome composition by improving salivary quality and quantity.^11^ Finally, although some evidence suggests that acidogenic probiotics may influence enamel hardness due to acid production,^19^ the protective balance achieved through microbiota modulation and host immune enhancement likely dominates under regular dietary exposure. Collectively, these findings justify the biological plausibility of our observed association between MedHi live dietary microbes and reduced root caries risk.^18^

### Dietary and Systemic Factors in Root Caries Risk

Dietary quality plays a pivotal role in maintaining oral and systemic health.^17^ In our model, higher vegetable intake was associated with a lower likelihood of root caries, consistent with studies linking comprehensive dietary quality scores (eg, HEI2015) to reduced caries risk.^12,23^Conversely, frequent consumption of sugar-sweetened beverages was positively associated with root caries, supporting prior evidence^3^ that simple sugar intake promotes acid production, fosters lactobacilli proliferation, and decreases salivary buffering capacity.^7^ Systemic conditions such as type 2 diabetes further exacerbate root caries risk,^24^ possibly through alterations in salivary flow rate and the oral microbiome.^4^ Meanwhile, it is important to emphasise not only oral hygiene practices like toothbrushing and the use of fluoridated products.^8^ These findings reinforce that root caries is a multifactorial disease influenced by diet, microbial ecology, and host metabolic status.

### Clinical and Public Health Implications

The current findings underscore the potential of MedHi live dietary microbes and probiotic-rich foods as accessible preventive strategies for root caries. Incorporating these foods into daily diets could complement established oral hygiene practices such as toothbrushing and fluoride use. Moreover, our machinelearning prediction model can facilitate early identification of individuals at elevated risk, allowing for personalised dietary guidance and preventive intervention in community and clinical settings.

### Strengths and Limitations

The major strength of this study lies in integrating nutritional, microbial, and systemic health data within a nationally representative sample, applying rigorous machinelearning methods. Nevertheless, several limitations should be acknowledged. The cross-sectional design precludes causal inference. Dietary intake derived from self-reported NHANES data may introduce recall bias. Furthermore, periodontal parameters^20,22^ and salivary indicators^25^ – important in root caries pathogenesis – were unavailable in the 2015–2020 data set, and thus could not be incorporated into the model. Future longitudinal studies are warranted to validate our findings and further elucidate the dynamic relationship between oral microbiome modulation, diet, and root caries development.

## CONCLUSION

In summary, a higher intake of MedHi live dietary microbes was associated with reduced prevalence of root caries. The machine‑learning-based risk model developed in this study may serve as a novel tool for early identification and prevention of root caries in older adults. Our results highlight the value of probiotic‑rich dietary patterns as part of a comprehensive approach to maintaining oral health.

### Acknowledgements

#### Ethical approval

The NCHS Ethics Review Board examined and approved this study. In order to take part in this study, the patients/participants gave their written informed consent.

#### Consent to participate

Not applicable.

#### Consent to publish

Not applicable.

#### Competing interests

The authors declare no competing interests.

#### Artificial intelligence use disclosure

The authors used an AI-assisted language tool (ChatGPT) exclusively to improve the clarity and grammar of the English text. All scientific content, including the design of the study, data analysis, interpretation of results, and final conclusions, was conceived and validated solely by the authors. The authors carefully reviewed and edited all AI-assisted text to ensure accuracy and originality.

#### Funding

This study was Supported by SanmingProject of Medicine in Shenzhen (No. SZSM202311009) and Shenzhen Natural Science Foundation (JCYJ20250604142420028).

## Appendix

**Fig 4:**
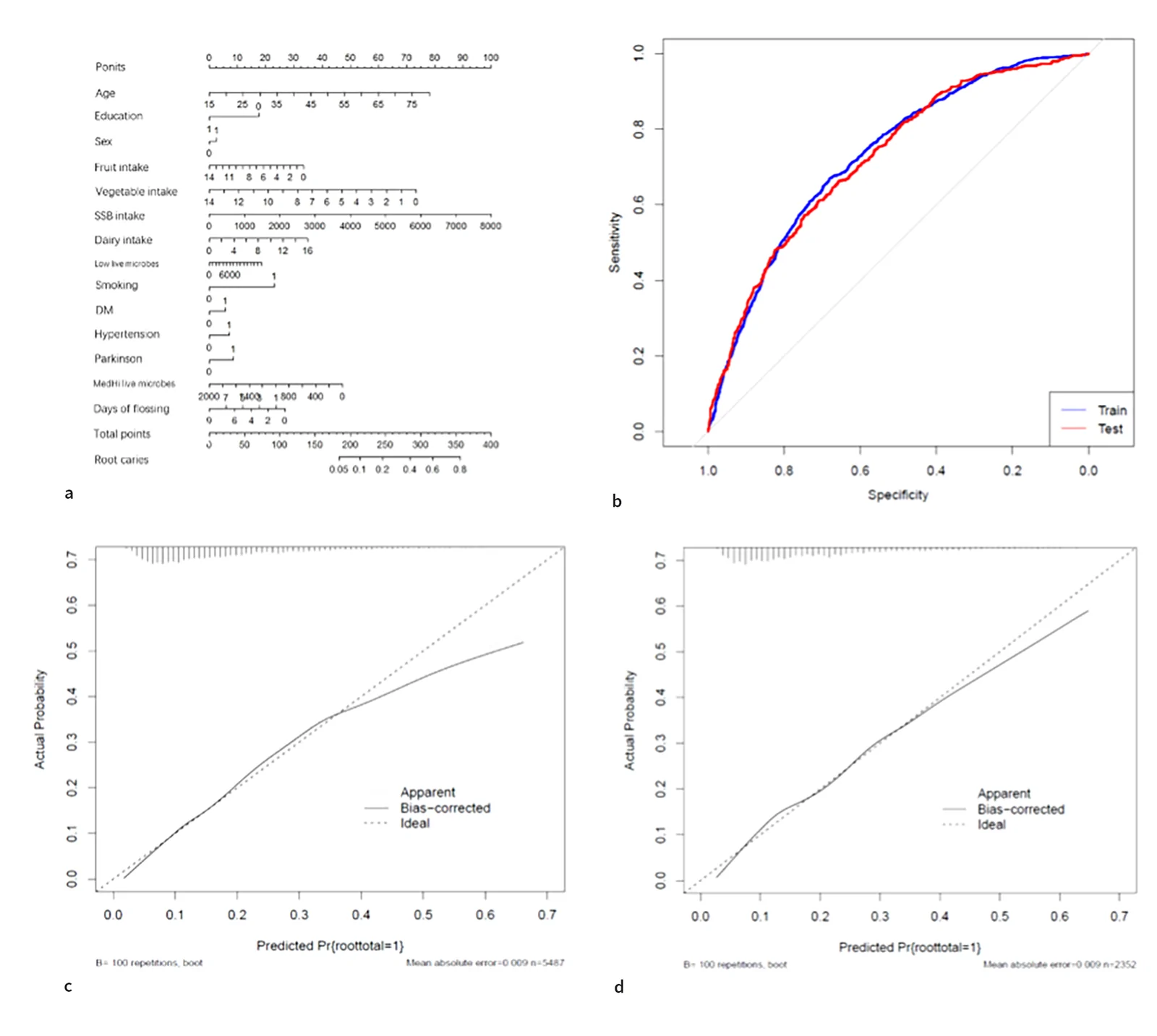
Nomogram and internal performance of the sensitivityanalysis model based on the λmin LASSO solution. *(a) *Nomogram of the 15-predictor logistic regression model derived from the λmin LASSO solution. *(b) *Receiver operating characteristic (ROC) curves for the training and testing data sets. *(c, d) *Calibration plots for the training *(c) *and testing *(d)* data sets, respectively. The diagonal dashed line represents perfect calibration, whereas the solid line indicates the performance of the 15predictor model; closer agreement between the two lines reflects better calibration. This sensitivity-analysis model showed discrimination and calibration comparable to those of the primary 9predictor model, based on the λ_{1SE} solution.

## References

[ref1] AlQranei MS, Balhaddad AA, Melo MAS (2020). The burden of root caries: updated perspectives and advances on management strategies. Gerodontology.

[ref2] Bashir NZ (2022). Update on the prevalence of untreated caries in the US adult population, 2017–2020. J Am Dent Assoc.

[ref3] Bernabé E, Vehkalahti MM, Sheiham A, Aromaa A, Suominen AL (2014). Sugar-sweetened beverages and dental caries in adults: a 4-year prospective study. J Dent.

[ref4] Cena JA, Reis LG, de Lima AK, Vieira Lima CP, Stefani CM, Dame-Teixeira N (2023). Enrichment of acid-associated microbiota in the saliva of type 2 diabetes mellitus adults: a systematic review. Pathogens.

[ref5] Chen DE, Kim N, Kim J-R, Yoo D, Oh D-H (2020). Microbial etiology and prevention of dental caries: exploiting natural products to inhibit cariogenic biofilms. Pathogens.

[ref6] Estruch R, Lamuela-Raventós RM (2023). Cardiovascular benefits of fermented foods and beverages: still up for debate. Nat Rev Cardiol.

[ref7] Faine MP, Allender D, Baab D, Persson R, Lamont RJ (1992). Dietary and salivary factors associated with root caries. Spec Care Dentist.

[ref8] Giacaman RA (2018). Sugars and beyond. The role of sugars and the other nutrients and their potential impact on caries. Oral Dis.

[ref9] Homayouni RA, Pourjafar H, Mirzakhani E (2023). A comprehensive review of the application of probiotics and postbiotics in oral health. Front Cell Infect Microbiol.

[ref10] Huang X, Kang L, Bi J (2025). Epidemiology of oral health in older adults aged 65 or over: prevalence, risk factors and prevention. Aging Clin Exp Res.

[ref11] Kaibori Y, Yamashita K, Nagakubo D (2023). The altered production and property of saliva induced by ingesting fermented food ingredients affect the oral microbiome composition in mice. Biosci Biotechnol Biochem.

[ref12] Kaye EA, Sohn W, Garcia RI (2020). The Healthy Eating Index and coronal dental caries in US adults. J Am Dent Assoc.

[ref13] Lingfang S, Zhongxin Z, Qiqi T, Libo H (2023). Association of interdental cleaning and untreated root caries in adults in the United States of America. Int Dent J.

[ref14] Lodi CS, Oliveira LV, Brighenti FL, Delbem ACB, Martinhon CCR (2015). Effects of probiotic fermented milk on biofilms, oral microbiota, and enamel. Braz Oral Res.

[ref15] Marco ML, Hutkins R, Hill C, Fulgoni VL, Cifelli CJ, Gahche J (2022). A classification system for defining and estimating dietary intake of live microbes in US adults and children. J Nutr.

[ref16] Mendonça FHBP, Dos Santos SSF, De Faria IDS, Silva CRGE, Leão MVP (2012). 2012. Effects of probiotic bacteria on Candida presence and IgA anti-Candida in the oral cavity of elderly. Braz Dent J.

[ref17] Moynihan P (2005). The interrelationship between diet and oral health. Proc Nutr Soc.

[ref19] Saha S, Chopra A, Kamath SU, Kashyap NN (2023). Can acid produced from probiotic bacteria alter the surface roughness, microhardness, and elemental composition of enamel? An in vitro study. Odontology.

[ref20] Sakurai I, Mayanagi G, Yamada S, Takahashi N (2023). In situ detection of endogenous proteolytic activity and the effect of inhibitors on tooth root surface. J Dent.

[ref21] Sivamaruthi BS, Kesika P, Chaiyasut C (2020). A review of the role of probiotic supplementation in dental caries. Probiotics Antimicrob Proteins.

[ref22] Suzuki S, Onose Y, Yoshino K, Takayanagi A, Kamijo H, Sugihara N (2020). Factors associated with development of root caries in dentition without root caries experience in a 2-year cohort study in Japan. J Dent.

[ref23] Tanaka K, Miyake Y, Sasaki S, Ohya Y, Matsunaga I, Yoshida T (2007). Relationship between intake of vegetables, fruit, and grains and the prevalence of tooth loss in Japanese women. J Nutr Sci Vitaminol (Tokyo).

[ref24] Vieira L, Pedrosa C, Chagas LFA, Marques RCR, Grisi DC, Salles LP (2023). 2023. Can hyperglycemia be associated with caries activity and root caries in adults. Prim Care Diabetes.

[ref25] Yas BA (2023). Interactive effect of salivary protein carbonyl, total glutathione, pH, and flow rate on root caries severity: a case–control study. J Int Soc Prev Commun Dent.

